# A comparison of complete mitochondrial DNA sequences of *Mnais costalis* Selys, 1869 (Odonata: Calopterygidae) from three different populations (one allopatric and two sympatric)

**DOI:** 10.1080/23802359.2019.1667888

**Published:** 2019-09-19

**Authors:** Hisashi Okuyama, Takuya Kiyoshi, Jun-Ichi Takahashi, Yoshitaka Tsubaki

**Affiliations:** a Faculty of Life Sciences, Kyoto Sangyo University, Kyoto, Japan;; b Department of Zoology, National Museum of Nature and Science, Tokyo, Japan;; c Center for Ecological Research, Kyoto University, Kyoto, Japan

**Keywords:** Next generation sequence, damselfly, *Mnais costalis*, allopatric population, sympatric population

## Abstract

In Japan, two closely-related damselflies, *Mnais costalis* Selys, 1869 (Odonata: Calopterygidae) and *M. pruinosa* Selys-Longchamps (Odonata: Calopterygidae), 1853, coexist, and they exhibit geographic variations in wing color, body size, and habitat preference. In this study, we analyzed the complete mitochondrial genome of *M. costalis* from Saga Prefecture, Japan (sympatric populations that exhibit wing color polymorphism), and compared the genome with *M. costalis* that exhibit monomorphic orange wing color. The mitochondrial genome of *M. costalis* from Saga Prefecture was identified as a circular molecule of 15,488 bp, similar to that found in other *M. costalis* populations. It was predicted to contain 13 protein-coding (PCG), 22 tRNA, and two rRNA genes, along with one A + T-rich control region. Among the PCGs, *ATP8* and *ATP6*, *ATP6* and *COIII*, *ND4* and *ND4L*, and *ND6* and *Cytb* shared seven, one, seven, and one nucleotides, respectively. The initiation codon ATG was found in eight genes, ATC in four, and ATT in one, while the termination codons TAA, TAG, TA, and T were observed in seven, one, two, and three genes, respectively. All the tRNA genes possessed a cloverleaf secondary structure, except for *tRNA-His* that lacks the TΨC loop. The average AT content of mitochondrial genome was 66.06%. From a phylogenetic analysis, the loss of wing color polymorphism in monomorphic sympatric populations is likely to occur with the coexistence of two *Mnais* species.

*Mnais costalis* Selys, 1869 (Odonata: Calopterygidae) often coexists with the closely related damselfly, *M. pruinosa* Selys 1853. These species exhibit geographic variations of wing color, body size, and habitat preference (e.g. Suzuki and Tamaishi 1982; Nomakuchi et al. 1984; Siva-Jothy and Tsubaki 1989; Okuyama et al. 2013; Tsubaki and Okuyama 2016). In allopatric populations, *M. costalis* males exhibit wing color polymorphism (Tsubaki et al. 1997), with orange morphs and clear morphs being observed. In sympatric populations of two *Mnais* species in the Chugoku, Shikoku, and Kyusyu regions of Japan, *M. costalis* males exhibit the same wing color polymorphism as allopatric populations, but are monomorphically orange-winged in the Chubu and Kinki regions. In this study, we analyzed the complete mitochondrial genome of *M. costalis* from a sympatric population in Kyusyu, and compared the genome with *M. costalis* from a sympatric population in Kinki (AP017642; Okuyama and Takahashi 2017), and from an allopatric population (KU871065; Lorenzo-Carballa et al. 2016).

We collected adult orange-winged male *M. costalis* individuals from Saga Prefecture, Japan in May 2017. The adult damselflies were transferred immediately to 99.5% ethanol. The specimen was stored the National Museum of Nature and Science, Japan (accession number: NSMT-I-Od-33337). Genomic DNA isolated from one damselfly was sequenced using the Illumina MiSeq platform (Illumina, San Diego, CA, USA). The complete mitochondrial genome of *M. costalis* (AP017642) was used as a reference sequence. The resultant reads were assembled and annotated using the MITOS web server (Bernt et al. 2013) and Geneious R9 (Biomatters). These 13 protein-coding genes (PCGs) and two rRNA genes sequences were aligned using MEGA6 (Tamura et al. 2013). The phylogenetic analysis was performed with the maximum likelihood (ML) criterion using TREEFINDER (Jobb 2011).

We succeeded in sequencing the entire mitochondrial genome of *M. costalis* from Saga Prefecture (DDBJ accession number AP019627). The genome consisted of a closed loop 15,488 bp-long, which included 13 PCGs, 22 tRNA genes, two rRNA genes, and one AT-rich control region, which resembles the genomic organization common in *M. costalis*. The heavy strand was predicted to have nine protein-coding and 14 tRNA genes, while the light strand was predicted to contain four protein-coding, eight tRNA, and two rRNA genes. Eight PCGs started with *ATG*; the *ND2*, *ATP8*, *ND3*, and *ND6* genes with ATC; and the *ND5* gene with ATT. Seven PCGs used the stop codon TAA; the *ND5* gene used TAG; the *COIII* and *ND4* genes used the incomplete stop codon TA; and the *COII*, *ND5*, and *Cytb* genes used a single T. All the tRNA genes possessed a cloverleaf secondary structure, except for *tRNA-His*. A phylogenetic analysis was constructed using 13 PCGs across 14 Zygoptera taxa (Figure 1). The phylogenetic relationships of three *M. costalis* populations suggested that the loss of wing color polymorphism in the Chubu and Kinki regions may have occurred after coexistence with *M. pruinosa*. However, this relationship is supported with a lower bootstrap value, therefore it is necessary to undertake further analysis of *M. costalis* mitochondrial genome data from these three distinct populations.

**Figure 1.**
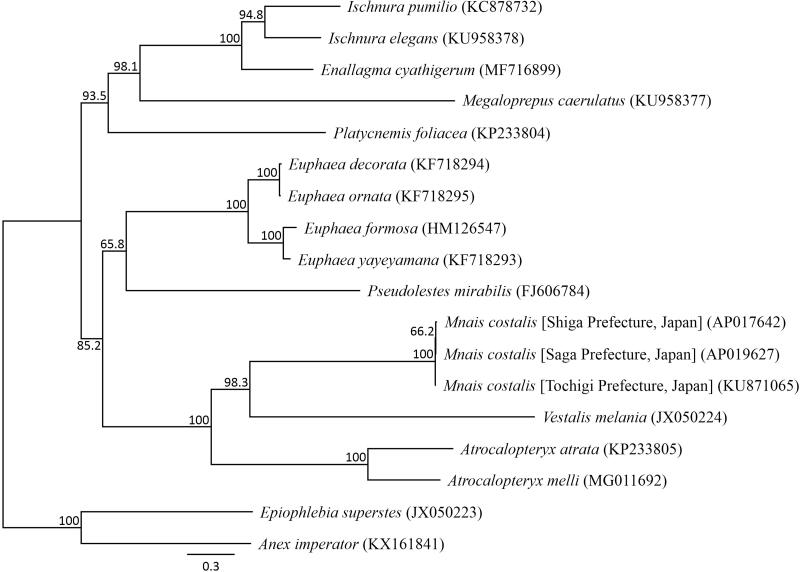
Phylogenetic relationships (maximum likelihood) of the Zygoptera based on the nucleotide sequences of the 13 protein-coding genes of the mitochondrial genome. Sequences from *Epiophlebia superstes* (JX050223, Wang et al. 2015) and *Anax imperator* (KX161841, Feindt et al. 2016) were used as an outgroup. These sequences were separated by codon positions, and for each partition, the optimal models of sequence evolution were used in the maximum likelihood method using TREEFINDER, based on the corrected Akaike information criterion. The numbers at the nodes indicate the bootstrap support inferred from 1000 bootstrap replicates. Alphanumeric terms indicate the DNA Database of Japan accession numbers.
